# Holocene melting of the West Antarctic Ice Sheet driven by tropical Pacific warming

**DOI:** 10.1038/s41467-022-30076-2

**Published:** 2022-05-20

**Authors:** Adam D. Sproson, Yusuke Yokoyama, Yosuke Miyairi, Takahiro Aze, Rebecca L. Totten

**Affiliations:** 1 Atmosphere and Ocean Research Institute, The University of Tokyo, Kashiwa, Japan; 2 Biogeochemistry Research Center, Japan Agency for Marine-Earth Science and Technology, Yokosuka, Japan; 3 Department of Earth and Planetary Sciences, Graduate School of Science, The University of Tokyo, Tokyo, Japan; 4 Graduate Program on Environmental Sciences, Graduate School of Arts and Sciences, The University of Tokyo, Tokyo, Japan; 5 Research School of Physics, The Australian National University, Canberra, ACT 02000 Australia; 6 Department of Geological Sciences, The University of Alabama, Tuscaloosa, AL United States

**Keywords:** Cryospheric science, Palaeoclimate

## Abstract

The primary Antarctic contribution to modern sea-level rise is glacial discharge from the Amundsen Sea sector of the West Antarctic Ice Sheet. The main processes responsible for ice mass loss include: (1) ocean-driven melting of ice shelves by upwelling of warm water onto the continental shelf; and (2) atmospheric-driven surface melting of glaciers along the Antarctic coast. Understanding the relative influence of these processes on glacial stability is imperative to predicting sea-level rise. Employing a beryllium isotope-based reconstruction of ice-shelf history, we demonstrate that glaciers flowing into the Amundsen Sea Embayment underwent melting and retreat between 9 and 6 thousand years ago. Despite warm ocean water influence, this melting event was mainly forced by atmospheric circulation changes over continental West Antarctica, linked via a Rossby wave train to tropical Pacific Ocean warming. This millennial-scale glacial history may be used to validate contemporary ice-sheet models and improve sea-level projections.

## Abstract (web summary)

Ice loss from the Amundsen Sea sector of West Antarctica is rapidly accelerating. Here, the authors reveal that this region also underwent thinning and retreat from 9 to 6 thousand years ago, due to atmospheric connections with a warming tropical Pacific.

## Introduction

Increasing ice loss and sea-level contribution from Antarctica since the early twenty-first century is caused by rapid thinning, retreat, and acceleration of major outlet glaciers of the West Antarctic Ice Sheet (WAIS)^1^. Recent WAIS mass loss has been focused within the Amundsen Sea Embayment (ASE), which may indicate the beginning of marine ice sheet instability^2,3^. Ice mass loss since the 1990s is attributed to the ocean-driven melting of ice shelves by the upwelling of relatively warm Circumpolar Deep Water (CDW) onto the West Antarctic continental shelf^1,4^. Precipitation and warming over West Antarctica was also more elevated in the 1990s than at any time over the last 200 years, linked to higher tropical Pacific Ocean temperatures^5,6^. Atmospheric rivers (narrow bands with enhanced water vapour flux) associated with this tropical-polar teleconnection represent 100% of summer surface melt in Marie Byrd Land^7^, presenting an additional contribution to global sea-level rise from the atmospheric-driven melting of glaciers along the West Antarctic coast. Significant uncertainty therefore remains for the permanence of increasing ice loss, projected contributions to sea level, and the dominant driving mechanisms behind mass balance change^1^. Integrated models predicting the timing and rate of future Antarctic ice mass loss rely on validation from long-term (10^2^–10^3^ yr) records of past ice-sheet change^8,9^, and therefore require glacial reconstructions since the Last Glacial Maximum (LGM)^10^.

**Fig. 1:**
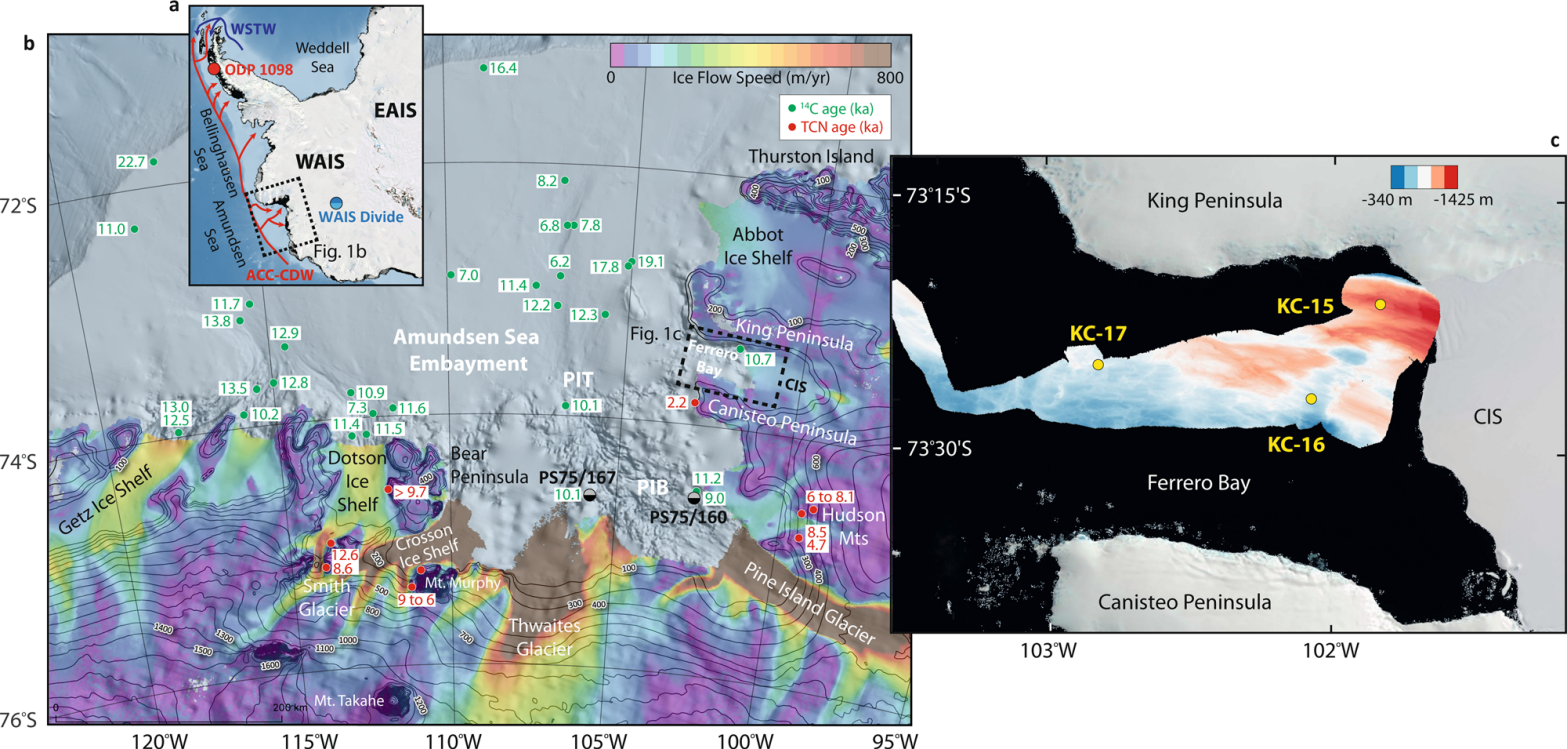
Maps of West Antarctica, the ASE, and Ferrero Bay. **a** Map of Antarctica displaying a generalised position of Antarctic Circumpolar Current-Circumpolar Deep Water (ACC-CSW) and Weddell Sea Transitional Water (WSTW). **b** Ice velocity^64,65^, satellite imagery^66^, radiocarbon dates and terrestrial cosmogenic nuclide dates^11,13,14,48^ for the Amundsen Sea Embayment, Pine Island Trough (PIT), and Pine Island Bay (PIB). **c** Location of OSO-0910 KC-15, KC-16, and KC-17 in Ferrero Bay with multibeam swath bathymetry^16^. Maps were constructed using Quantarctica from the Norwegian Polar Institute^67^.

Marine records from the ASE reveal that the Amundsen sector of the WAIS (Fig. 1a) reached its modern limits by the Early Holocene^11^ (Fig. 1b), driven by upwelling CDW onto the continental shelf due to a southerly position of the Southern Hemisphere westerly wind belt^12^. However, the mechanism behind subsequent early-to-mid-Holocene thinning of ice streams surrounding the ASE^13,14^ is still not well understood^15^. Here we apply beryllium (Be) isotopes, a proxy for glacial processes, to provide a 10.3 kyr history of the Cosgrove Ice Shelf (CIS) in the eastern ASE (Fig. 1b). We measured the reactive ^10^Be abundance ([^10^Be]_reactive_), ^9^Be abundance ([^9^Be]_reactive_) and ^10^Be/^9^Be ratios in marine sediments collected during the *IB Oden* Southern Ocean cruise (OSO-0910)^16^ (“Methods”). Kasten cores KC-15, KC-16 and KC-17 were collected just offshore the modern terminus of the CIS in Ferrero Bay (Fig. 1c). Beryllium isotope records are supported by published multi-proxy analysis of total organic carbon (TOC), total nitrogen (TN), microfossil abundance and assemblages (diatoms and foraminifera), and magnetic susceptibility (MS), which together provide a record of Holocene productivity and grounding line retreat^17^. Beryllium isotope records from KC-15 are compiled onto a new age-depth model (Fig. S1) to elucidate the timing of CIS retreat during the Holocene.

## Results and discussion

**Fig. 2:**
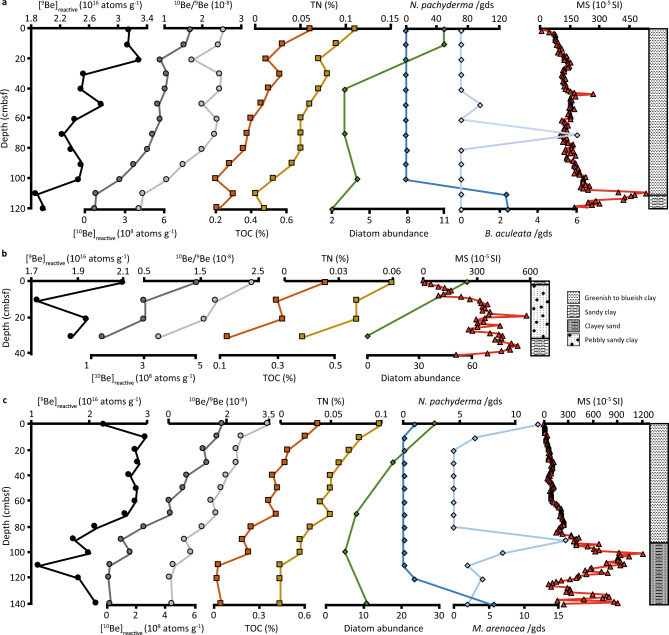
Proxy data from Ferrero Bay. **a**–**c** The reactive ^9^Be abundance, ^10^Be abundance, and ^10^Be/^9^Be ratios (this study), alongside total nitrogen (TN), total organic carbon (TOC), diatom abundance, foraminifera taxa (counts per gram of dried sediment, gds), magnetic susceptibility (MS), and lithological descriptions^17^ of OSO-0910 KC-15 (**a**), KC-16 (**b**), and KC-17 (**c**).

**Fig. 3:**
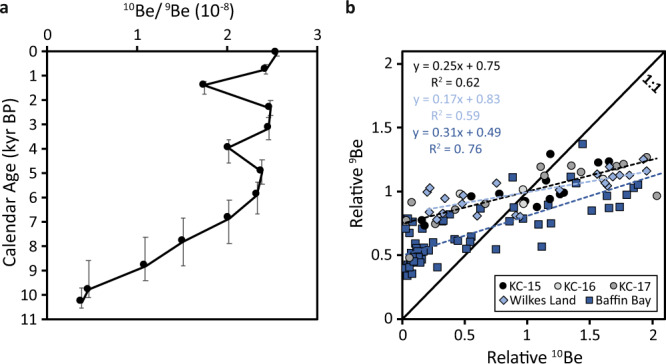
Ice-shelf history of the CIS for the Holocene. The ^10^Be/^9^Be ratios (**a**) and relative ^10^Be vs. ^9^Be relationship (**b**) in OSO-0910 Kasten core KC-15. Error bars represent 2σ confidence intervals (Fig. S1). Relative Be isotope values from offshore Wilkes Land^37^ and Baffin Bay^36^ are presented using light blue diamonds and dark blue squares, respectively. The dashed black line, light blue line, and dark blue line represent the relationship for samples from this study, Wilkes Land^37^, and Baffin Bay^36^, respectively.

**Fig. 4:**
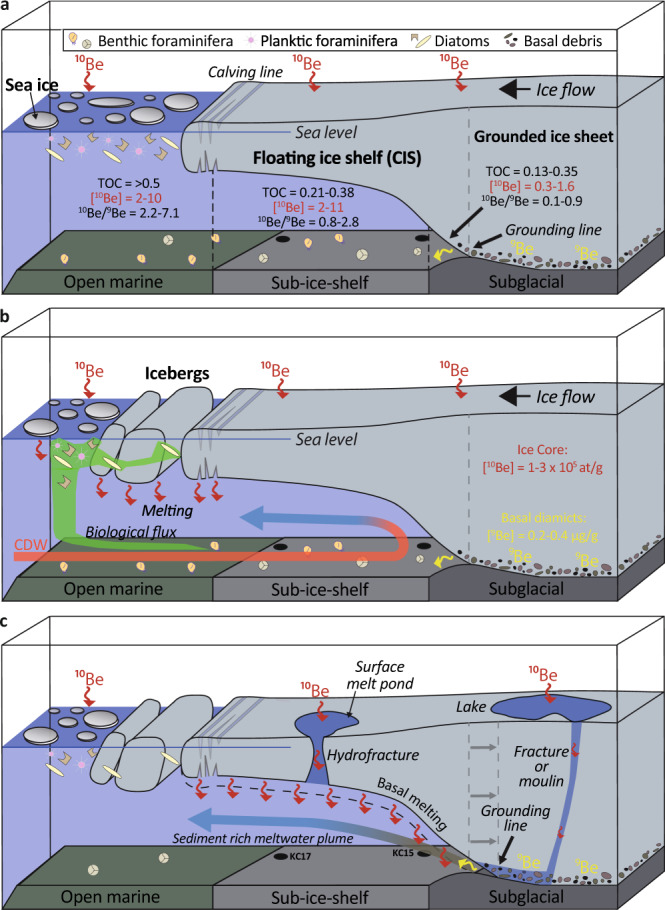
Illustration of the potential processes influencing Be isotopes within Ferrero Bay. **a** Open marine, sub-ice-shelf, and subglacial environments are defined by different geochemical compositions^27,34^. **b** Melting of icebergs and sea ice near the calving line releases ^10^Be which is rapidly scavenged by diatom frustules and organic matter before becoming advected under the ice-shelf with other marine material by ocean currents such as intruding CDW. **c** Meltwater rich in ^10^Be is released from the ice-shelf base and scavenged by fine-grained sediment delivered from the grounding line. This figure was modified from Yokoyama et al.^24^ and White et al.^27^.

The [^10^Be]_reactive_, [^9^Be]_reactive_, and ^10^Be/^9^Be ratios recorded for KC-15, KC-16, and KC-17 are presented in Fig. 2. Cores KC-15 and KC-17 display four key features: (1) from the base of the cores to ~111 cm below the seafloor (cmbsf), the [^10^Be]_reactive_, [^9^Be]_reactive_, ^10^Be/^9^Be, TOC, TN, and diatom abundance are relatively low, while MS and the abundance of the calcareous planktic foraminifera, *Neogloboquadrina pachyderma*, are relatively high (Fig. 2a, c); (2) between ~111 and ~71 cmbsf, [^10^Be]_reactive_, ^10^Be/^9^Be, TOC, and TN increases, diatom abundance remains low, MS decreases, and *N. pachyderma* becomes absent. Reactive ^9^Be also increases between ~111 and ~71 cmbsf in KC-17 (Fig. 2c) yet plateaus in KC-15 above ~101 cmbsf (Fig. 2a); (3) between ~71 and ~31 cmbsf, [^10^Be]_reactive_, [^9^Be]_reactive_, ^10^Be/^9^Be, TOC, TN, and MS remain relatively constant while the presence of the benthic foraminifera, *Bulimina aculeata*, is observed in KC-15 (Fig. 2a, c); (4) above ~31 cmbsf, TOC, TN, and diatom abundance increases and MS gradually declines (Fig. 2).

The strong correlation between [^10^Be]_reactive_ and ^10^Be/^9^Be ratios in all three cores (Tables S3–S5) suggests the rise in [^10^Be]_reactive_ is largely controlling variation in the ^10^Be/^9^Be ratios. Reactive ^10^Be, and therefore ^10^Be/^9^Be, displays a similar trend to TOC and TN in KC-15, KC-16, and KC-17 (Tables S3–S5). Magnetic susceptibility records a negative correlation to Be isotopes (Tables S3–S5), representing a lithologic transition from coarse-grained sediments with high sand content (% sand = 14–46%) at the base of the core to overlying fine-grained sediments that are rich in silt and clay^17^. Diatom abundance correlates with Be isotopes in KC-15 and KC-17, with the exception of [^9^Be]_reactive_ in KC-17, however, diatoms were not counted at the same sampling interval making a direct comparison with Be concentration difficult (Tables S3, S5).

The sediments from KC-15 record the last ~10.3 kyr of glacial history for Ferrero Bay and the CIS. According to our new age model (Fig. S1; Table S1), Be isotope data reveal three periods of interest (Fig. 3a): (1) in the Early Holocene, between ~10.3 and ~9.8 kyr BP, the ^10^Be/^9^Be ratios are low at ~0.42; (2) transitioning from the Early Holocene to Mid-Holocene, ^10^Be/^9^Be increases between ~9.8 kyr BP and ~5.9 kyr BP from ~0.46 to ~2.33; (3) then through the Late Holocene, ^10^Be/^9^Be ratios are relatively constant at ~2.3. During the Late Holocene, ^10^Be, ^9^Be, TOC, and TN potentially display millennial-scale variability, increasing after ~1.4 kyr BP (Fig. 2), but higher temporal resolution would be needed to support this notion. In the following section, we discuss the environmental and physical factors that could contribute to Be isotope variation in Ferrero Bay during the Holocene.

### Beryllium isotope systematics in glacial environments

The cosmogenic isotope ^10^Be is produced by the interaction of cosmic rays with oxygen and nitrogen in the atmosphere and is deposited to the Earth’s surface via dust or precipitation^18^, becoming enriched in surface waters under high precipitation such as the equatorial Pacific (~1100 atoms/g)^19,20^. Beryllium-9, on the other hand, is released during the chemical weathering of silicate minerals^18^ and is enriched in waters near high fluvial or aeolian inputs e.g., equatorial Atlantic surface waters in proximity to Saharan dust plumes^20^. Away from regions of high surface inputs, such as the South Pacific, Be follows a general profile of nutrient scavenging with a depletion in the surface mixed layer (~750 atoms/g) and an enrichment in deeper waters (~2000 atoms/g), reflecting the high scavenging potential of Be^19–21^. The ^10^Be/^9^Be of Pacific seawater has an average ratio of ~1 × 10^−7^ but ranges from ~0.4 × 10^−7^ to ~3.1 × 10^−7^ in equatorial surface waters of the Atlantic and Pacific due to additional inputs of ^9^Be and ^10^Be, respectively^20^. The disparate sources of ^10^Be and ^9^Be led to fluctuations in the ^10^Be/^9^Be of seawater, recorded in marine sediments and ferromanganese crusts, over Quaternary climate cycles, due to large regional variability in fluvial inputs between glacial and interglacial periods^22^.

The Be isotope composition of marine sediments on the continental shelf is controlled by the relative mixing of two reactive phases, one sourced from the coast, defined by low ^10^Be/^9^Be_reactive_ due to the input of fluvial ^9^Be, and another sourced from entraining seawater, defined by high ^10^Be concentrations and ^10^Be/^9^Be ratios^23^. The high ^10^Be/^9^Be of seawater is augmented in polar regions by the input of ^10^Be from atmospheric sources^18,24^ and/or the melting of sea ice ([^10^Be] = ~0.25–0.65 ×10^4^ atoms/g)^25^ and ice sheets ([^10^Be] = ~1–3 × 10^5^ atoms/g)^26^, which act to accumulate meteoric ^10^Be overs years to millennia, leading to exceptionally high surface water ratios which have been observed in the Deep Canadian Basin (~1.4 × 10^−7^)^25^ and Drake Passage (~3.3 × 10^−7^)^19,20^, respectively. The export of meltwater signals further offshore may be enhanced by the calving of icebergs and their subsequent passage off the continental shelf^27^.

The separate transport pathways and sources of ^10^Be and ^9^Be in ice sheets has allowed Be in glaciomarine sediments to become a powerful proxy of glacial dynamics^24,27–31^. Open marine sediments close to the calving line of ice shelves are defined by higher Be concentrations and ^10^Be/^9^Be ratios relative to other glacial environments due to efficient scavenging of Be by diatoms from seawater defined by higher overall ratios as previously discussed^24,28^. Lower ^10^Be concentrations and ^10^Be/^9^Be ratios can be found in sub-ice shelf sediments, induced by lower productivity and limited advection of open marine material into the ice-shelf cavity^24,27,28^. Finally, sediments in proximity to the grounding line receive ^9^Be from the basal zone during glacial erosion leading to exceptionally low ^10^Be/^9^Be ratios^27,31^. Here, we relate Be isotope variation in Ferrero Bay to three main processes: (1) environmental setting; (2) depositional processes; and (3) meltwater contribution (Fig. 4).

### The Cosgrove Ice Shelf in the Holocene

Bathymetric surveys reveal that grounded ice occupied Ferrero Bay during the LGM as part of the larger ice stream in the Cosgrove-Abbot Trough, which reached the continental shelf edge north of the Abbot Ice Shelf^11,32,33^ (Fig. 1b). The Amundsen Sea Ice Shelf retreated into the Pine Island Trough (PIT) to a position just north of Ferrero Bay by ~13.6 kyr BP, before moving south of Ferrero Bay to ~150 km from the current grounding line of Pine Island Glacier by ~10.6 kyr BP^32^. The grounded ice sheet within Ferrero Bay retreated landward at a similar time from the mouth of Ferrero Bay at ~13.6 kyr BP^32^ to the proximal core site of KC-15 by ~11 kyr BP^17^, maintaining a floating ice shelf that reached the distal core site of KC-17^17,33^. Based on the updated age model for Ferrero Bay sediment cores, the changes in lithology and magnetic susceptibility in KC-15 (KC-17) indicate two phases of grounding line retreat within Ferrero Bay terminating by 124 (131) and 105 (99) cmbsf, corresponding to ~10.7 kyr BP and ~9.6 kyr BP, respectively^17^.

Prior to ~9.8 kyr BP (>111 cmbsf in KC-15), low Be concentrations and ^10^Be/^9^Be indicate that the KC-15 and KC-17 core sites remained proximal to the grounding line. Subsequently, an increasing trend in [^10^Be]_reactive_ and ^10^Be/^9^Be is observed between ~9.8 kyr BP (~111 cmbsf) and ~5.9 kyr BP (~71 cmbsf) suggesting a gradual retreat of the grounding line and the development of a more distal environmental setting (Figs. 2a, c, 3a and 4a). The grounding line stabilised, and a permanent ice shelf established in Ferrero Bay between ~5.9 kyr BP (~71 cmbsf) and ~2.3 kyr BP (~31 cmbsf), as indicated by relatively constant ^10^Be/^9^Be of ~2.1 (Figs. 2a, c, 3a and 4a). Finally, the ice-shelf collapsed and open marine conditions established by ~2.3 kyr BP at the site of KC-17 and ~1.4 kyr BP at the site of KC-15. The progression of glacial retreat and changing environments is supported by a shift from a coarse-grained lithology with low TOC and high MS near the base of the sediment record, to a fine-grained lithology with intermediate TOC and a gradually decreasing MS up-section, and, finally, to TOC values above 0.5% and higher abundance of diatoms and benthic foraminifera, indicating open marine conditions^17,34^ (Figs. 2 and 4a).

The relative change in Be concentration across these various glacial environments is likely to be controlled by depositional processes such as dilution, scavenging efficiency, and/or extraction efficiency^22^, much like the percentage of TOC^35^. From the bottom of the cores to ~111 cmbsf in KC-15 and KC-17, TOC is likely diluted by the input of coarse-grained clastic material from the grounding line with a high sand content of 27–46% (ref. ^17^). From ~111 cmbsf, TOC is largely controlled by steadily increasing accumulation rates (calculated from the new age-depth model) of fine-grained (% sand ≦ 12.5%) sediment up-section, which allows for a more rapid passage of organic carbon through the near-surface zone, reducing its degradation^35^. Reactive ^10^Be, and to a lesser extent ^9^Be, displays a strong correlation to TOC (Fig. 2; Tables S3–S5), suggesting Be concentration may also be largely controlled by dilution and/or scavenging efficiency. A similar lithological control is observed in Baffin Bay whereby [Be]_reactive_ and ^10^Be/^9^Be are relatively low over the last glacial cycle due to the poor scavenging efficiency of coarse-grained carbonate-rich material but Be isotopes increase by over an order of magnitude during ice-surging events associated with the production of fine-grained feldspar-rich glacial flour^36^. The percentage of TOC and Be concentration in Ferrero Bay sediments may also be related to productivity (i.e., diatom and foraminifera abundance) in the overlying water column or the advection of biogenic material into the ice-shelf cavity. However, the diatoms were not counted at the same sampling interval and other organic plankton such as dinoflagellates are not included in previous studies^17^, limiting a direct comparison between TOC, Be abundance, and productivity.

Much like Be isotope concentration, the relationship between relative ^10^Be and relative ^9^Be is largely controlled by extraction efficiency, scavenging efficiency, and/or dilution, generally defined by a 1:1 relationship. Changes in the gradient of this relationship above or below one is driven by an additional input of ^9^Be or ^10^Be, respectively^22,37^. The relative ^10^Be vs. relative ^9^Be relationship of sediments from Baffin Bay and offshore Wilkes Land are defined by a gradient less than one (Fig. 3b) controlled by the additional flux of meltwater-derived ^10^Be from nearby ice sheets^36,37^. This leads to higher ^10^Be/^9^Be ratios during periods of intense glacial discharge such as Heinrich events^36^ and Pliocene interglacials^37^. Sediments from KC-15 to KC-17 display a similar relationship to Baffin Bay and offshore Wilkes Land (Fig. 3b) indicating an additional input of meltwater-derived ^10^Be to Ferrero Bay may be contributing to the increase in ^10^Be/^9^Be ratios from ~9.8 kyr BP to ~5.9 kyr BP (Fig. 3a).

Meltwater input to Ferrero Bay is derived from one of two sources: (1) the calving of ice shelves and melting of icebergs, the meltwater of which is subsequently advected under the CIS (Fig. 4b), or (2) the basal and/or surface melting of the CIS (Fig. 4c). From 10.3 to 9.8 kyr BP, limited productivity of diatoms and calcareous foraminifera resulted from the advection of offshore currents under the Amundsen Sea ice shelf, as evidenced by low benthic productivity and the presence of the planktic foraminifer *N. pachyderma*, suggesting that ocean currents accessed the grounding line at this time^17,33^ (Fig. 2a, c). During this period, [^10^Be]_reactive_ and ^10^Be/^9^Be are at their lowest, however—indicating a negligible amount of Be is advected from the calving line or from offshore currents (Fig. 4b).

From 9.8 to 2.3 kyr BP, diatom abundance is low, and calcareous foraminifera and benthic productivity are low but present, indicative of a distal glacio-marine environment and limited advection under the extended paleo-ice shelf^17,33^ (Fig. 2a, c). During the period, [^10^Be]_reactive_ and ^10^Be/^9^Be increase (Fig. 2a, c), indicating that the majority of Be is not sourced by water flowing from the continental shelf and is more likely associated with subglacial water flowing out from beneath the ice sheet (Fig. 4c). This is supported by observations from Lake Maruwan Oike, East Antarctica, which records peak [^10^Be]_reactive_ and ^10^Be/^9^Be during a brackish transition to lacustrine conditions, implying Be is derived from the melting of local glaciers^29^. Importantly, a benthic foraminifera species thought to be associated with CDW, *B. aculeata*, is present at 51 and 71 cmbsf in KC-15 (Fig. 2a), indicating CDW incursion during the Mid-Holocene^17^, but the advection of this offshore water mass apparently had limited influence over Be isotope values which remain high but relatively constant during this time.

To summarise, Be isotope data from Ferrero Bay suggests the CIS underwent a dramatic shift from the Early-to-Mid-Holocene: (1) the grounding line was proximal to KC-15 prior to ~9.8 kyr BP during the Early Holocene, with a permanent ice-shelf extending beyond KC-17 into the ASE; (2) the grounding line retreated from the Early-to-Mid-Holocene between ~9.8 kyr BP and ~5.9 kyr BP, associated with melting of the CIS and its paleo-ice stream; (3) the grounding line and the CIS remained stable for the rest of the Mid-Holocene until ~2.3 kyr BP; (4) the CIS retreated past KC-15 to its modern position during the Late Holocene, establishing open marine conditions in Ferrero Bay. In the following section, we relate our findings to the wider context of the Amundsen Sea sector of the WAIS and the possible mechanisms driving West Antarctic climatic change during the Holocene.

### Atmospheric warming led to Holocene melting

**Fig. 5:**
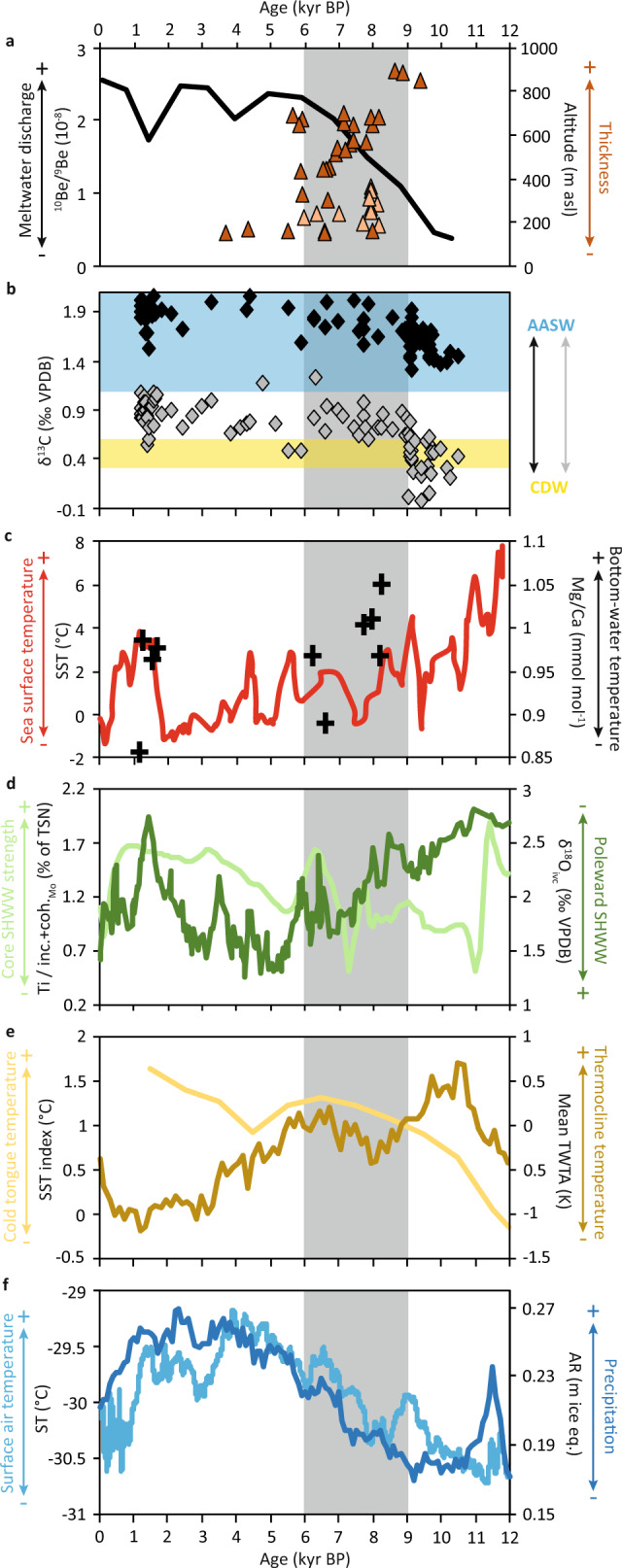
Proxy records of ASE forcing mechanisms during the Holocene. **a** Meltwater discharge from the CIS based on ^10^Be/^9^Be ratios from OSO-0910 KC-15 (black line) alongside the thinning history of the Pine Island (light orange triangles) and Pope (dark orange triangles) glaciers derived from exposure ages of samples from Mt. Murphy and Mt. Moses/Maish Nunatak, respectively^13,14^. **b** *δ*^13^C values of benthic (grey diamonds) and planktic (black diamonds) foraminifera relative to estimated values for the CDW (yellow box) and AASW (blue box) from PS75/160 and PS75/167 (Fig. 1b)^12^. **c** Five-point moving average of sea surface temperature (SST) estimated for the Palmer Deep, western Antarctic Peninsula shelf (red line; ODP 1098, Fig. 1a) and Mg/Ca ratios of benthic foraminifera as a semi-quantitative representation of bottom-water temperatures within PIB (black crosses; PS75/160, Fig. 1b)^12,38^. **d** Titanium µ-XRF analysis from Emerald Lake on Macquarie Island^39^ (light green line) and five-point moving average of ice volume corrected *Globorotalia inflata* *δ*^18^O (*δ*^18^O_ivc_) recorded in the South Atlantic^40^ (dark green line). **e** Alkenone SST index from the eastern Pacific equatorial cold tongue^41^ (light yellow line) and mean thermocline water temperature anomaly of the Indo-Pacific warm pool^43^ (dark yellow line). **f** Atmospheric temperature^68^ (light blue line) and accumulation rates^46^ (dark blue line) for the WAIS Divide core (Fig. 1a). The grey box highlights the interval of 9 to 6 cal kyr BP.

Melting and retreat of the CIS and its paleo-ice stream recorded here corresponds well to a respective ~560 m and ~150 m thinning of the Pope Glacier^18^ and Pine Island Glacier^16^ from 9 to 6 cal kyr BP and 8 to 6 cal kyr BP, indicating a widespread melting event within the Amundsen Sea sector of the WAIS (Fig. 5a). Early Holocene deglaciation of PIB is linked to the enhanced upwelling of relatively warm CDW (Fig. 5c) into the ASE^12,33^ (Fig. 5b). Benthic *δ*^13^C data from PIB records a rapid reduction of CDW inflow at 9 kyr BP while planktic *δ*^13^C data display a gradual decline between 10.4 and 8 kyr BP (Fig. 5b), associated with a lessening of CDW heat supply^12,38^ (Fig. 5c). Sediments studied here likely correspond to “Unit 1” and “Facies 3” in cores from PIT^33^ and PIB^32^, respectively, representing a switch from proximal glacimarine sediments accessed by warm water flowing onto the continental shelf before ~9.6 kyr BP, as evidenced by the presence of *N. pachyderma* (Fig. 2a, c)^17^, to distal meltwater-derived glacimarine sediments, devoid of biogenic material, emanating from beneath the ice sheet by ~7.8 ka BP^33^. The ^10^Be/^9^Be ratios from KC-15 are at their lowest during maximum CDW incursion into the PIB region, increasing after 9 kyr BP (Fig. 5a, b). This suggests that early-to-mid-Holocene retreat of the CIS was generally not associated with the upwelling of CDW into Ferrero Bay, albeit some contribution is constrained to the Mid-Holocene between ~5.9 and ~4 kyr BP as inferred by the presence of the CDW-associated benthic foraminifer *B. aculeata*^17^ (Fig. 2a).

During the Holocene, the Southern Hemisphere westerly winds were at their weakest between 11 and 9 cal kyr BP, gradually intensifying during the Mid-Holocene to maximum values at ~ 5 cal kyr BP^39^, associated with a poleward migration of the westerly wind belt between 8.5 and 5.5 cal kyr BP^40^ (Fig. 5d). The southerly shift in the westerly winds coincides with a progressive warming of the tropical Pacific^41^ (Fig. 5e) and greater thermal contrast across the zone of strong westerlies^42^. A steeper west to east upper-ocean temperature gradient and intensified Walker circulation in the equatorial Pacific led to a second peak in thermocline warming of the Indo-Pacific warm pool during the Mid-Holocene, reaching peak warming between 7 and 6 cal kyr BP^43^ (Fig. 5e). Tropical Pacific warming can generate an atmospheric Rossby wave train that influences atmospheric circulation over the Amundsen Sea, leading to advection of warm air onto continental West Antarctica (i.e., Ellsworth Land and Marie Byrd Land), associated with cooling and greater sea-ice formation around the Antarctic Peninsula^5^, suggesting a link between Pacific warming and ice sheet thinning around the ASE^7,44^.

A southerly position of the westerly jet and an increase in atmospheric moisture content led to a poleward displacement and a higher frequency of Pacific atmospheric rivers during the Mid-Holocene^45^. A strengthening of the Amundsen Sea Low increased cyclonic-driven precipitation over continental West Antarctica between 9 and 6 kyr BP, as evidenced by a relatively small surface air temperature warming coupled to a dramatic increase in accumulation rates at the WAIS Divide ice core^46^ (Fig. 5f). Intense rainfall would have enhanced surface melting over the West Antarctica coast, thus decreasing surface albedo and increasing regional melt, while higher mixed-phase cloud cover would have reduced radiative cooling and therefore meltwater refreezing^7,44^. Furthermore, if atmospheric rivers travel perpendicular to coastal mountain ranges near the Amundsen Sea, the resulting föhn winds can enhance surface melt through adiabatic warming of descending dry air^7^.

Surface melting and/or surface-meltwater-enhanced calving of floating ice shelves would trigger rapid ice flow accelerations in outlet glaciers^9^, leading to retreat of the CIS and meltwater discharge of ^10^Be, via basal melting or meltwater injection from surface melt ponds and lakes through ice fractures and moulins^47^, and thinning of ice streams along the Ross and Amundsen Sea coasts^13,14,48,49^ during the Early-to-Mid-Holocene (Figs. 4c and 5a). Conversely, an ice core from the east Antarctic Peninsula records a cooling trend from 10 to 8 cal kyr BP^50^, associated with a reduction in glacial discharge^51^ and greater sea-ice formation^52^, while sediment core biomarkers suggest surface water cooling^38^ (Fig. 5c) along the west Antarctic Peninsula between 9 and 6 cal kyr BP. The cooling trend observed along the Antarctic Peninsula at the same time as a warming trend in the ASE is consistent with a teleconnection between tropical Pacific warming and atmospheric circulation over West Antarctica^5^. Hence, we suggest that higher tropical Pacific Ocean sea surface temperatures led to atmospheric warming and higher precipitation over continental West Antarctica, inducing melting and retreat of the CIS and thinning of other glaciers in the ASE between 9 and 6 cal kyr BP.

### Outlook and future research

Presently, glaciers flowing into the ASE are the main Antarctic contribution to global sea-level rise, averaging 0.23 ± 0.02 mm yr^−1^ between 1992 and 2013 (ref. ^2,3^). The driving mechanism of sea-level rise acceleration has been attributed to ocean-driven melting of ice shelves that buttress glacial flow^1,4,12^. However, atmospheric rivers associated with tropical-polar teleconnections present a mechanism for substantial contribution to seasonal surface melt over continental West Antarctica as well^7^. Greater atmospheric moisture content and poleward expansion of the Hadley cells under rising greenhouse gas emissions suggest atmospheric river climatology is already beginning to resemble that of the Mid-Holocene^45^, possibly linked to extensive melt events along the Ross and Amundsen Sea coasts since the 1990s^6,7,44^. Our study suggests that future warming and increasing precipitation brought by enhanced atmospheric river activity could contribute to further surface melting and significant glacial discharge into the ASE. This information is vital for validating numerical models, thereby improving future predictions of sea-level rise^8,9,53,54^ which is currently estimated to be as much as 1 m from Antarctica by the end of the twenty-first century^9^. Here, we demonstrate that Be isotopes can be successfully employed alongside other sediment and biological proxies to reconstruct past changes in depositional environment, ice shelf extent, and meltwater input—a parameter that is completely missing from most glacial records since the LGM^11,27^. However, uncertainty remains owing to the multiple sources and processes influencing the Be isotopes systematics of glacimarine sediments^27^. Future work will need to constrain the Be abundance in sea ice, ice sheets, and shelf waters, whilst also determining how Be is incorporated into sediments from the grounding line to the continental slope.

## Methods

### Study location and core sampling

Ferrero Bay is located within eastern PIB in the ASE (Fig. 1b), reaching a maximum depth of ~1300 m to the north and an average of ~700 m to the south near the Canisteo Peninsula (Fig. 1c). The King Peninsula separates Ferrero Bay from the Abbot Ice Shelf. Ferrero Bay connects to the continental shelf edge through the eastern trough of the ASE, receiving relatively warm and saline CDW from PIB below ~275 m water depth^17^. Three Kasten cores were collected from Ferrero Bay during the *IB Oden* OSO-0910 expedition from 2009 to 2010: KC-15 (73.36° S, 101.84° W; 1274 m water depth), a 1.3 m sediment core from the innermost fjord; KC-16 (73.45° S, 102.08° W; 706 m water depth), a 0.4 m sediment core from a structural high; and, KC-17 (73.42° S, 102.83° W; 855 m water depth), a 1.4 m core recovered from the outer bay (Fig. 1c). All three cores generally consist of clays ranging from clayey sand to greenish-blueish clay (Fig. 2) and record local glacial history, biological productivity, and oceanographic conditions during the Holocene^17^. KC-15 and KC-17 are divided into four units based on sedimentological, geochemical, and palaeontological properties with KC-16 including only the uppermost unit^17^.

### Beryllium isotope analysis

Reactive Be ([^10^Be]_reactive_ and [^9^Be]_reactive_) was separated from marine sediments using the technique previously presented^29^. Approximately 0.1 g of sediment was dried and crushed before being leached with 0.04 M hydroxylamine hydrochloride in 25% acetic acid for 7 h at 80 °C. An aliquot of the leached solution was measured for reactive ^9^Be using a Thermo® ELEMENT XR high resolution inductively coupled plasma mass spectrometer (HR-ICP-MS) at the Atmosphere and Ocean Research Institute (AORI), the University of Tokyo (UTokyo), after spiking with 5 µl of indium (1 µg/g) solution^55^. The remaining solution was spiked with 1 ml of a 0.097 mg/ml ^9^Be carrier before purification using two solvent extractions of acetylacetone in the presence of EDTA followed by precipitation of Be(OH)_2_ with NH_4_ (ref. ^56,57^). The resulting hydroxide was converted to BeO powder using a microwave ceramic crucible^58^ before being mixed with niobium, inserted into a copper cathode, and measured by a National Electrostatic Corporation accelerator mass spectrometer (AMS) at the UTokyo Micro Analysis Laboratory, Tandem Accelerator (MALT)^59^.

### Age-depth model

Radiocarbon analysis of two marine carbonate samples^33^ and seven bulk samples^17^ were previously obtained from KC-15. We provide a new age-depth model (Fig. S1) based on the modelling routine, *Undatable*, which uses the Bayesian ^14^C calibration software, *MatCal*, to take into account analytical uncertainty associated with ^14^C measurements and depth uncertainties of 2 cm (ref. ^60^). Carbon-14 ages were corrected for local effects^17,61^ and a marine reservoir effect (Δ*R*) of 900 ± 100 years (ref. ^62^) prior to calibration using the Marine13 curve^63^. *Undatable* was run for 10^6^ iterations using a bootstrapping percentage of 20% and a Gaussian SAR uncertainty factor of 0.1 (ref. ^60^).

## Supplementary information

Supplementary Information

## Data Availability

All data generated in this study are included in the Supplementary information file.
