# Supplementary material for: Holocene melting of the West Antarctic Ice Sheet driven by tropical Pacific warming
Source: Nat Commun. 2022 May 20;13:2434. doi: 10.1038/s41467-022-30076-2 (PMC9123186; doi:10.1038/s41467-022-30076-2)
Supplement: Supplementary file 1 — Supplementary Information [file 41467_2022_30076_MOESM1_ESM.pdf]

**Supplementary Information:**

# Holocene melting of the West Antarctic Ice Sheet driven by tropical Pacific warming


**Adam D. Sproson[1,2*], Yusuke Yokoyama[1,2,3,4,5], Yosuke Miyairi[1], Takahiro Aze[1] and Rebecca L. Totten[6]**

[1]Atmosphere and Ocean Research Institute, The University of Tokyo, Kashiwa, Japan.
[2]Biogeochemistry Research Center, Japan Agency for Marine-Earth Science and Technology, Yokosuka, Japan.
[3]Department of Earth and Planetary Sciences, Graduate School of Science, The University of Tokyo, Tokyo, Japan
[4]Graduate Program on Environmental Sciences, Graduate School of Arts and Sciences, The University of Tokyo, Tokyo, Japan
[5]Research School of Physics, The Australian National University, Canberra, ACT 02000, Australia
[6]Department of Geological Sciences, The University of Alabama, Tuscaloosa, AL, USA

Corresponding author: Adam Sproson (adamsproson@gmail.com)


| Depth (cm) | Mid-depth (cm) | Calendar age (cal yr BP) | $[^{10}Be]_{reactive}$ ($10^8$ atoms $g^{-1}$) | 2σ | $[^{9}Be]_{reactive}$ ($10^{16}$ atoms $g^{-1}$) | 2σ | $^{10}Be/^{9}Be$ ($10^{-8}$) | 2σ |
|---|---|---|---|---|---|---|---|---|
| *KC-15* | | | | | | | | |
| 0-2 | 1 | $61^{+140}_{-104}$ | 8.05 | 0.71 | 3.16 | 0.19 | 2.55 | 0.37 |
| 10-12 | 11 | $750^{+183}_{-205}$ | 7.61 | 0.51 | 3.14 | 0.05 | 2.43 | 0.20 |
| 20-22 | 21 | $1403^{+367}_{-152}$ | 5.75 | 1.07 | 3.30 | 0.07 | 1.74 | 0.36 |
| 30-32 | 31 | $2332^{+274}_{-331}$ | 6.27 | 0.50 | 2.53 | 0.11 | 2.48 | 0.31 |
| 40-42 | 41 | $3153^{+475}_{-460}$ | 6.14 | 0.56 | 2.50 | 0.01 | 2.46 | 0.23 |
| 50-52 | 51 | $3964^{+631}_{-323}$ | 5.59 | 1.11 | 2.78 | 0.08 | 2.01 | 0.46 |
| 60-62 | 61 | $4941^{+514}_{-502}$ | 5.73 | 0.61 | 2.40 | 0.01 | 2.38 | 0.26 |
| 70-72 | 71 | $5898^{+748}_{-521}$ | 5.23 | 0.39 | 2.24 | 0.14 | 2.33 | 0.32 |
| 80-82 | 81 | $6852^{+1024}_{-742}$ | 4.77 | 0.43 | 2.36 | 0.01 | 2.02 | 0.19 |
| 90-92 | 91 | $7813^{+978}_{-986}$ | 3.78 | 0.60 | 2.51 | 0.02 | 1.51 | 0.25 |
| 100-102 | 101 | $8804^{+589}_{-1173}$ | 2.68 | 0.44 | 2.46 | 0.05 | 1.09 | 0.20 |
| 110-112 | 111 | $9784^{+335}_{-1197}$ | 0.86 | 0.28 | 1.88 | 0.02 | 0.46 | 0.15 |
| 120-122 | 121 | $10274^{+247}_{-578}$ | 0.75 | 0.31 | 1.98 | 0.01 | 0.38 | 0.16 |

**Table S1.** Depth, calendar age, and beryllium isotope measurements for KC-15.

| Depth (cm) | Mid-depth (cm) | $[^{10}Be]_{reactive}$ ($10^8$ atoms $g^{-1}$) | 2σ | $[^{9}Be]_{reactive}$ ($10^{16}$ atoms $g^{-1}$) | 2σ | $^{10}Be/^{9}Be$ ($10^{-8}$) | 2σ |
|---|---|---|---|---|---|---|---|
| *KC-16* | | | | | | | |
| 0-2 | 1 | 5.08 | 0.72 | 2.10 | 0.07 | 2.42 | 0.43 |
| 10-12 | 11 | 3.06 | 0.89 | 1.73 | 0.01 | 1.77 | 0.53 |
| 20-22 | 21 | 3.05 | 0.75 | 1.94 | 0.01 | 1.57 | 0.39 |
| 30-32 | 31 | 1.46 | 0.56 | 1.88 | 0.02 | 0.78 | 0.31 |
| 50-52 | 51 | 6.59 | 0.97 | 2.78 | 0.06 | 2.37 | 0.40 |
| *KC-17* | | | | | | | |
| 0-2 | 1 | 7.82 | 1.05 | 2.27 | 0.19 | 3.45 | 0.75 |
| 10-12 | 11 | 7.52 | 0.99 | 2.98 | 0.12 | 2.52 | 0.43 |
| 20-22 | 21 | 6.59 | 0.96 | 2.81 | 0.02 | 2.35 | 0.36 |
| 30-32 | 31 | 6.81 | 0.83 | 2.86 | 0.04 | 2.38 | 0.33 |
| 40-42 | 41 | 5.49 | 0.74 | 2.71 | 0.03 | 2.03 | 0.29 |
| 50-52 | 51 | 5.19 | 0.73 | 2.83 | 0.01 | 1.84 | 0.26 |
| 60-62 | 61 | 4.25 | 1.02 | 2.81 | 0.04 | 1.51 | 0.38 |
| 70-72 | 71 | 4.38 | 0.87 | 2.64 | 0.02 | 1.66 | 0.34 |
| 80-82 | 81 | 2.54 | 0.76 | 2.12 | 0.03 | 1.20 | 0.37 |
| 90-92 | 91 | 1.01 | 0.44 | 1.75 | 0.01 | 0.58 | 0.25 |
| 100-102 | 101 | 1.62 | 0.56 | 2.02 | 0.05 | 0.80 | 0.30 |
| 110-112 | 111 | 0.22 | 0.23 | 1.13 | 0.02 | 0.19 | 0.21 |
| 120-122 | 121 | 0.11 | 0.20 | 1.83 | 0.02 | 0.06 | 0.11 |
| 140-142 | 141 | 0.27 | 0.24 | 2.15 | 0.05 | 0.13 | 0.12 |

**Table S2.** Depth and beryllium isotope measurements for KC-16 and KC-17.

| | $[^{9}Be]_{reac}$ | $[^{10}Be]_{reac}$ | $^{10}Be/^{9}Be$ | TN | TOC | Diatom abundance | MS | n |
|---|---|---|---|---|---|---|---|---|
| $[^{9}Be]_{reac}$ | 1 | | | | | | | 13 |
| $[^{10}Be]_{reac}$ | 0.79 | 1 | | | | | | 13 |
| $^{10}Be/^{9}Be$ | 0.57 | 0.95 | 1 | | | | | 13 |
| TN | 0.82 | 0.97 | 0.87 | 1 | | | | 13 |
| TOC | 0.73 | 0.89 | 0.77 | 0.94 | 1 | | | 13 |
| Diatom | 0.96 | 0.77 | 0.55 | 0.83 | 0.81 | 1 | | 6 |
| MS | -0.71 | -0.91 | -0.89 | -0.89 | -0.71 | -0.78 | 1 | 13 |

**Table S3.** Pearson (*r*) correlation coefficients for KC-15.

| | $[^9Be]_{reac}$ | $[^{10}Be]_{reac}$ | $^{10}Be/^9Be$ | TN | TOC | MS | n |
|---|---|---|---|---|---|---|---|
| $[^9Be]_{reac}$ | 1 | | | | | | 4 |
| $[^{10}Be]_{reac}$ | 0.63 | 1 | | | | | 4 |
| $^{10}Be/^9Be$ | 0.48 | 0.98 | 1 | | | | 4 |
| TN | 0.50 | 0.98 | 0.99 | 1 | | | 4 |
| TOC | 0.60 | 0.99 | 0.98 | 0.99 | 1 | | 4 |
| MS | -0.74 | -0.98 | -0.93 | -0.92 | -0.95 | 1 | 4 |

**Table S4.** Pearson (*r*) correlation coefficients for KC-16.

| | $[^9Be]_{reac}$ | $[^{10}Be]_{reac}$ | $^{10}Be/^9Be$ | TN | TOC | Diatom abundance | MS | n |
|---|---|---|---|---|---|---|---|---|
| $[^9Be]_{reac}$ | 1 | | | | | | | 14 |
| $[^{10}Be]_{reac}$ | 0.80 | 1 | | | | | | 14 |
| $^{10}Be/^9Be$ | 0.71 | 0.98 | 1 | | | | | 14 |
| TN | 0.70 | 0.97 | 0.99 | 1 | | | | 14 |
| TOC | 0.75 | 0.97 | 0.98 | 0.99 | 1 | | | 14 |
| Diatom | 0.19 | 0.80 | 0.84 | 0.82 | 0.77 | 1 | | 5 |
| MS | -0.62 | -0.75 | -0.72 | -0.72 | -0.69 | -0.74 | 1 | 14 |

**Table S5.** Pearson (*r*) correlation coefficients for KC-17.

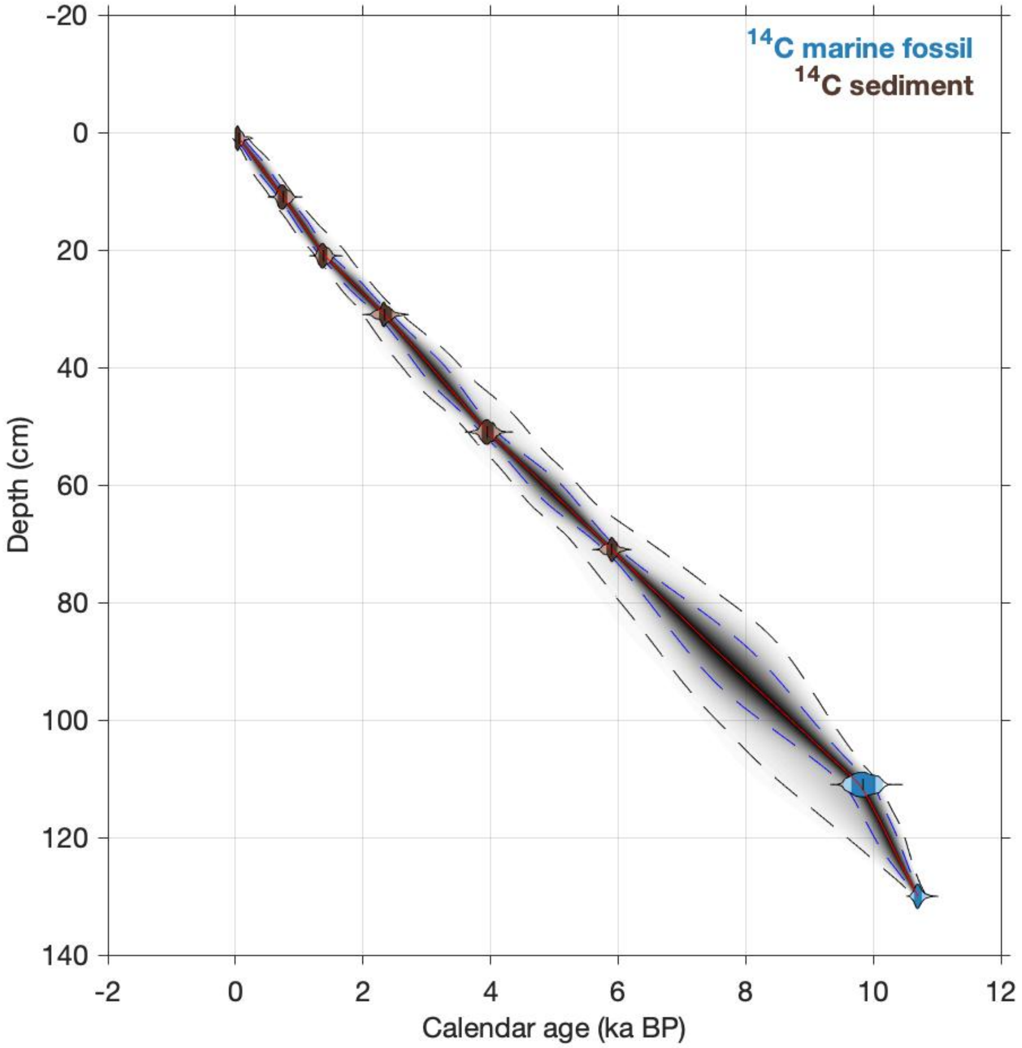


**Figure S1.** *Undatable* age-depth model for KC-15 based on organic carbon and marine carbonate $^{14}C$ dates from Minzoni *et al.*[1] and Kirshner *et al.*[2], respectively. The red line, blue broken line, and black broken line represents the median, 1σ confidence, and 2σ confidence intervals, respectively.

**Supplementary References**


1 Minzoni, R. T. *et al.* Oceanographic influences on the stability of the Cosgrove Ice Shelf, Antarctica. *The Holocene* **27**, 1645-1658, doi:10.1177/0959683617702226 (2017).

2 Kirshner, A. E. *et al.* Post-LGM deglaciation in Pine island Bay, west Antarctica. *Quaternary Science Reviews* **38**, 11-26 (2012).
